# Commentary: Mitigating the other pandemic

**DOI:** 10.1016/j.xjtc.2021.08.033

**Published:** 2021-08-25

**Authors:** Jahnavi Kakuturu, Charlotte Spear, J. W. Awori Hayanga

**Affiliations:** Department of Cardiovascular and Thoracic Surgery, WVU Heart and Vascular Institute, West Virginia University, Morgantown, WVa

**Figure:**
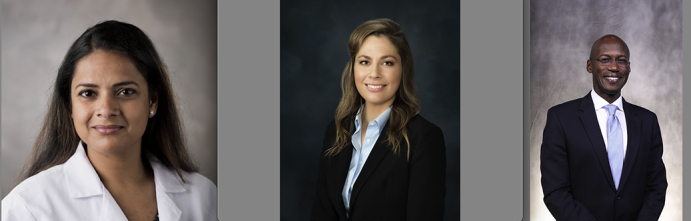
Jahnavi Kakuturu, MD, Charlotte Spear, MD, and J. W. Awori Hayanga, MD, MPH, FACS, FRCS, FCCP

**Central Message**

Navigating the challenge of obesity in extracorporeal support requires careful attention.

See Article page 335.

In this article, Javidfar and colleagues^1^ address the use of extracorporeal support in the patient with obesity. The article is timely and relevant in view of the ever-increasing proportion of patients with obesity requiring support for acute severe respiratory failure. Perhaps, more poignant, nevertheless, is the coincident backdrop of the coronavirus disease 2019 (COVID-19) pandemic. The disproportionate risk of obesity is manifest in the median body mass index of 33 in patients requiring extracorporeal membrane oxygenation (ECMO).^2^ A better understanding of the confluence of these 2 epidemics is therefore likely to be of value to the readership and the surgical community at large as they each grapple with an ever-increasing volume of patients with obesity requiring extracorporeal support.

Recent metanalysis has demonstrated that obesity may exacerbate COVID-19 by an increased expression of angiotensin-converting enzyme, to which the virus spike protein binds and renders the lungs susceptible to attack.^3^ Obesity is also associated with an exacerbation of inflammatory and immune responses that may induce an exaggerated inflammatory response.^4^ The independent risk, therefore, posed by obesity is certainly worthy of evaluation in view of the inherent challenges in cannulation, ventilatory management, and rehabilitation that characterize the care of this cohort of patients. Javidfar and colleagues^1^ provide pragmatic insight into the use of transpulmonary esophageal pressure measurement to guide titration of positive end expiratory pressure as well as in the use of unconventional cannulation strategies to optimize venous drainage. The authors deftly navigate the controversy regarding obesity as an independent risk factor for death on extracorporeal support. Indeed, this has been a source of contention in the ECMO literature for several years, and there is vociferous opinion both for and against the assertion that it is.^4^^,^^5^

It is plausible, nevertheless, that the greater potential for cannulation injury may be responsible for most of the excess mortality. In stark contradistinction, however, once on extracorporeal support, obesity may, nevertheless, proffer a survival advantage. Indeed, using the Predicting Death for Severe ARDS (PRESERVE) scoring to identify factors associated with death, for instance, one observes that obesity represents a 2-point reduction in risk. Ostensibly, therefore, this would favor a lower mortality in patients with obesity.^6^ The authors steer clear of any recommendation as to type or preferred technique of tracheostomy, which would have likely added pragmatic value to the narrative beyond their somewhat tempered recommendation for early tracheostomy. Obesity, nonetheless, continues to be a somewhat-enigmatic feature of extracorporeal support and bears with it a unique set of challenges equally as worthy of discussion as they are of mitigation.

## References

[1] Javidfar J., Zaaqoq A.M., Yamashita M.H., Eschun G., Jacobs J.P., Heinsar S. (2021). Venovenous extracorporeal membrane oxygenation in obese patients. J Thorac Cardiovasc Surg Tech.

[2] Barbaro R.P., MacLaren G., Boonstra P.S., Iwashyna T.J., Slutsky A.S., Fan E., Extracorporeal Life Support Organization (2020). Extracorporeal membrane oxygenation support in COVID-19: an international cohort study of the Extracorporeal Life Support Organization registry. Lancet.

[3] Csige I., Ujvárosy D., Szabó Z., Lőrincz I., Paragh G., Harangi M. (2018). The impact of obesity on the cardiovascular system. J Diabetes Res.

[4] Yang J., Tian C., Chen Y., Zhu C., Chi H., Li J. (2021). Obesity aggravates COVID-19: an updated systematic review and meta-analysis. J Med Virol.

[5] Simonnet A., Chetboun M., Poissy J., Raverdy V., Noulette J., Duhamel A., LICORN and the Lille COVID-19 and Obesity study group (2020). High prevalence of obesity in severe acute respiratory syndrome coronavirus-2 (SARS-CoV-2) requiring invasive mechanical ventilation. Obesity (Silver Spring).

[6] Schmidt M., Zogheib E., Rozé H., Repesse X., Lebreton G., Luyt C.E. (2013). The PRESERVE mortality risk score and analysis of long-term outcomes after extracorporeal membrane oxygenation for severe acute respiratory distress syndrome. Intensive Care Med.

